# A new era for the Journal of Animal Science and Technology

**DOI:** 10.1186/2055-0391-56-1

**Published:** 2014-05-15

**Authors:** Inho Choi

**Affiliations:** School of Biotechnology, Yeungnam University, Gyeongsan, South Korea

## Abstract

We anticipate that this new alliance between The Korean Society of Animal Science and Technology and BioMed Central will elevate our journal one step higher, and facilitate easier access for those overseas as well as domestic researchers, who in turn will support us to contribute updated research outcomes in this field. We cordially invite all of you to participate and submit the results of your research so that everyone can learn and strive towards the goals that we all share.

It is both a pleasure and honor to be writing this editorial on behalf of The Korean Society of Animal Science and Technology (KSAST), and to announce our partnership with the global publisher, BioMed Central, for the future publication of our journal.

Documentations of hunting animals as a source of meat pre-date the evolution of man to the era of the chimpanzees [1]. As humans evolved, so has their relationship with wild animals as they learned to domesticate and harness their physical abilities which exceed those of humans. Animal domestication, which is the process of developing the mutually useful relationship between animals and humans, has revolutionized our way of life. Even today in an age where technology is rapidly replacing traditional methods; many countries must rely on animals to sustain their way of living. From plowing farms and sources of transportation to use of trade for materials such as leather and wool, the role of domestic animals will continue to grow with future generations. In fact, the role of these companions is expanding even to our emotional needs as discoveries have been made of the correlation of animal companionships to curing mental or emotional disorders in children.

Among the numerous benefits provided by domesticated animals, the nutritional values obtained from sources with high quality protein (meat, egg, milk, etc.) required to maintain our health is one of the most significant. Consumption of animal-derived products generally tends to increase with the developmental status of the country’s economy. Currently, 30 percent of the total human demand for food and agriculture comes from livestock in forms such as meat, milk, eggs, and fertilizer for crops, while also serving as an imperative cash reserve in many farming systems. This large contribution to food security comes from approximately 5,000 types of breeds comprised from 40 domestic animal species. Increasing the breeding of livestock will be a key factor in overcoming food shortages in countries where consumption exceeds amount of available food. Thus, we can expect to see changes in livestock production in the decades to come.

Though there are many cases for the beneficial aspects of livestock, we should not refrain from mentioning the risks and importance of containing these risks. As animal trade has become a rewarding industry the magnitude of global movement of animals is overwhelming. Animals are legally imported for exhibitions at zoos, scientific education, conservation programs, food, tourism, etc. However, this globalization has increased the spread of infectious diseases and the risk of non-communicable diseases. Research in animal science reduces the possible spread of disease by investigating the physical health of the animals being imported or exported. Scientists suggest solutions that employ familiar tools such as post-arrival screening of animals using reliable laboratory tests, experiential treatment for known diseases, or isolation of the animals for an appropriate length of time. Despite these efforts little success has been made in dealing with diseases such as mad cow disease, the avian flu, foot and mouth disease, and others, which has had deathly consequences for animals and humans alike. Only through extensive research - utilizing the latest resources and technologies along with the collaboration of international scientists - will we contain these potential epidemics [2].

It is important to understand that domestic animals are no longer bound to specific regions or countries and are continuing to globally impact everyone. In this respect, nationwide collaborations and efforts to share the information obtained by researchers will play a leading role in accenting the beneficial aspects and minimizing harmful consequences. It is the goal of the *Journal of Animal Science and Technology* to initiate this transfer of information by reshaping its journal publication system into one that provides convenient availability of resources that can be shared.

Recent advents of biotechnology and its application to livestock research are rapidly shifting the classical pdigm of research in this field. For instance, genome research in farm animals has sped the understanding of functions in various organisms at the genomic level [3, 4]. This approach can also be applied to the identification of novel molecular signatures or predictive biomarkers which can aid in improving production efficiency in farm animals [5]. Moreover, results from the research of livestock provide scientists with a solid research model with which to study diseases in animals and humans [6, 7].

To enlighten other researchers of new information in this field, the *Journal of Animal Science and Technology* (*JAST*) initially launched in the year of 1956 and was renamed in 2000 to its current title. *JAST* is a peer-reviewed, open access, online journal that publishes original research, review articles, and notes in all fields of animal science. Topics covered by the journal range from classical livestock-oriented research to the most recent issues in animal biotechnology, animal behavior, environment, and welfare. In addition, research results on aquatic and wildlife species and laboratory animal species that address fundamental questions related to livestock and companion animal biology will also be considered for publication. *JAST* is thus dedicated to addressing those issues by publishing research-oriented views and results that aid in the betterment of animals as a whole.

We are certain that by associating with this prestigious publishing house, *JAST* will have greater visibility that will attract new authors from around the world to publish in our journal and gather new readers. The journal will be indexed in PubMed and Scopus, and published articles are archived in PubMed Central. In an attempt to continually improve the impact and competitiveness of this journal, manuscripts will receive immediate attention upon arrival. Submitted manuscripts will be handled by a Section Editor and evaluated by at least two independent reviewers who are either members of the Editorial Board or *ad hoc* referees. The reviewers, experts in the area to which the manuscript pertains, will critically analyse the manuscript in a timely manner. Acceptance for publication is based on the scientific content, originality, and presentation of the material. Accepted manuscripts will be published electronically within a few weeks of their final approval date.

The first articles in volume 56 of *JAST*, published by BioMed Central as open access manuscripts, are on cattle breeding and genetics [8], immunology in fish [9], and monogastric animal nutrition [10]. We welcome comments on articles to which an author may wish to reply. Article metrics will be provided for all articles to help readers assess the overall importance and impact of an article in terms of citations, downloads, as well as discussion and debate concerning the article.

## References

[1] Stanford CB (1996). The Hunting Ecology of Wild Chimpanzees: Implications for the Evolutionary Ecology of Pliocene Hominids. Am Anthropologist.

[2] Smith J, Sones K, Grace D, MacMillan S, Tarawali S, Herrero M (2013). Beyond milk, meat, and eggs: Role of livestock in food and nutrition security. Anim Front.

[3] Lee EJ, Malik A, Pokharel S, Ahmad S, Mir BA, Cho KY, Kim J, Kong JC, Lee DM, Chung KY, Kim SH, Choi I (2014). Identification of Genes Differentially Expressed in Myogenin Knock-down Bovine Muscle Satellite Cells During Differentiation Through RNA Sequencing Analysis. PLoS One.

[4] Lee KT, Chung WH, Lee SY, Choi JW, Kim J, Lim D, Lee S, Jang GW, Kim B, Choy YH, Liao X, Stothard P, Moore SS, Lee SH, Ahn S, Kim N, Kim TH (2013). Whole-genome resequencing of Hanwoo (Korean cattle) and insight into regions of homozygosity. BMC Genomics.

[5] Andersson L (2001). Genetic dissection of phenotypic diversity in farm animals. Nat Rev Genet.

[6] Park TS, Han JY (2012). *piggyBac* transposition into primordial germ cells is an efficient tool for transgenesis in chickens. PNAS.

[7] Lee EJ, Bhat AR, Kamli MR, Pokharel S, Chun T, Lee YH, Nahm SS, Nam JH, Hong SK, Yang B, Chung KY, Kim SH, Choi I (2013). Transthyretin Is a Key Regulator of Myoblast Differentiation. PLoS One.

[8] Lee SH, Park BH, Sharma A, Dang CG, Lee SS, Choi TJ, Choy YH, Kim HC, Jeon KJ, Kim SD, Yeon SH, Park SB (2014). Kang, HS: Hanwoo cattle: origin, domestication, breeding strategies and genomic selection. J Anim Sci Technol.

[9] Park KH, Choi S (2014). Effects of Prunella vulgaris labiatae extract on specific and non-specific immune responses in tilapia, (Oreochromis niloticus). J Anim Sci Technol.

[10] Mushtaq MMH, Parvin R, Kim J (2014). Carcass and body organ characteristics of broilers supplemented with dietary sodium and sodium salts under a phase feeding system. J Anim Sci Technol.

